# Stem Cell Therapy for Neurodegenerative Diseases: How Do Stem Cells Bypass the Blood-Brain Barrier and Home to the Brain?

**DOI:** 10.1155/2020/8889061

**Published:** 2020-09-04

**Authors:** Yvonne Cashinn Chia, Clarice Evey Anjum, Hui Rong Yee, Yenny Kenisi, Mike K. S. Chan, Michelle B. F. Wong, Shing Yi Pan

**Affiliations:** ^1^Baden R&D Laboratories GmbH, Germany; ^2^Baden Research and Testing (Asia Pac) Sdn Bhd, Malaysia

## Abstract

Blood-brain barrier (BBB) is a term describing the highly selective barrier formed by the endothelial cells (ECs) of the central nervous system (CNS) homeostasis by restricting movement across the BBB. An intact BBB is critical for normal brain functions as it maintains brain homeostasis, modulates immune cell transport, and provides protection against pathogens and other foreign substances. However, it also prevents drugs from entering the CNS to treat neurodegenerative diseases. Stem cells, on the other hand, have been reported to bypass the BBB and successfully home to their target in the brain and initiate repair, making them a promising approach in cellular therapy, especially those related to neurodegenerative disease. This review article discusses the mechanism behind the successful homing of stem cells to the brain, their potential role as a drug delivery vehicle, and their applications in neurodegenerative diseases.

## 1. Introduction

The blood-brain barrier (BBB) is a selective barrier formed by the central nervous system (CNS) endothelial cells (ECs) which are connected by continuous tight junctions, creating a restricted movement of molecules across the BBB. The physiological barrier is regulated by a series of physical, transport, and metabolic properties as well as interactions with different vascular, immune, and neural cells [1]. Apart from junction proteins, the BBB is comprised of adherence proteins, transporters, basal lamina, and extracellular matrix at the molecular level [2]. The BBB is surrounded by endothelial cells, pericytes, astrocytic foot processes, neurons, mast cells, microglia, and circulating immune cells [3–5].

The selective barrier capacity of BBB allows it to regulate CNS homeostasis and protect the CNS against toxins, pathogens, inflammation, injury, and disease [1, 6]. However, disruption or dysregulation of the BBB may cause changes in permeability that permit pathogens into the brain or cause neuroinflammation, consequently contributing towards the progression of several CNS-related diseases including neurodegenerative diseases (e.g., Parkinson's disease and Alzheimer's disease), cerebrovascular disease (e.g., stroke), and traumatic brain injuries [7–11].

In general, there are two pathways that allow molecular movement across the BBB: first, through free diffusion via lipid solubility and, second, through catalysed transport [12]. Through extensive studies, researchers have found that compounds that are fat-soluble and/or very small in sizes, such as alcohol and certain low molecular weight narcotics or hormones (i.e., below 400-500 Dalton), can easily pass through the ECs that make up the BBB via the transcellular pathway [13, 14]. In contrast, specific transporters or receptors are necessary to facilitate the movement of various other larger nutrients, ions, and macromolecules [15]. Due to the restrictive characteristic of the BBB, there is an obstacle for drug delivery to the brain. An estimated 98% of potential therapeutics for brain disorders fail to penetrate the BBB [16, 17]. In light of these challenges, efforts have been made to create strategies to modulate and bypass the BBB so that life-saving drugs can reach specific targets in the brain without disturbing other ongoing activities [18].

It is vital to understand the mechanism of BBB regulation in treating neurological diseases as many CNS therapeutics are unable to bypass the BBB. The use of stem cells to bypass the BBB for CNS-related disease treatment has been promising. While stem cells are most commonly delivered via direct transplantation which allows nonsystemic homing, they often cause injury at the site of injection. In contrast, the administration of stem cell through intravenous, intramuscular, or intranasal does not cause injury, but stem cells must systemically home and bypass the BBB. This review article further explains the homing mechanism of stem cells bypassing the BBB and the applications of stem cell therapy in neurodegenerative diseases.

## 2. How Do Stem Cells Pass Through the BBB and Home?

Many different stem cells can be used in the treatment of neurological diseases, including but not limited to mesenchymal, neural, and embryonic stem cells. For the sake of discussion in this review, we will be citing MSCs as an example, considering it is the most widely used stem cells in brain-related treatment. The ability to protect damaged tissue and differentiate into a range of cells by secreting trophic, immune-modulatory, or other engineered therapeutic factors makes them an excellent choice in cellular therapy [19–21]. Nevertheless, the BBB remains a major physical barrier that MSCs and other stem cells must overcome to reach their targeted site in the brain and exert their therapeutic effects [4]. Unfortunately, no detailed studies have been conducted yet on how much the BBB is an active barrier for MSCs in treating the brain [22].

However, studies have shown that MSCs are capable of migrating across endothelial cells by either the paracellular or transcellular pathway and subsequently preferentially home back to the site of inflammation or injury in the brain to exert their therapeutic effects [23, 24]. This has been demonstrated in a whole-body imaging study in mouse using MSC-labelled fluorescence magnetic nanoparticles [25]. While the mechanism of how endogenous MSCs migrate and function in response to injury remains poorly understood, certain injuries such as traumatic brain injury, stroke, brain tumour, or aging are believed to compromise the efficiency of BBB protection [8, 9, 26, 27]. This allows MSCs to migrate across the endothelial cells via paracellular pathways through the formation of a transient interendothelial gap [23]. It has been postulated that activation of endothelial cells and astrocytes in some of these CNS injuries causes lower tight junction integrity and formation of paracellular gaps which allow cell migration via the paracellular route [20]. In addition, MSCs are also capable of abolishing and splitting tight junctions between endothelial cells [24].

**Figure 1:**
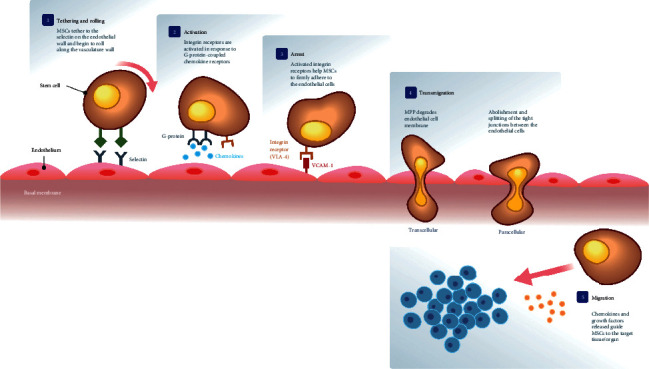
The multistep cascade of MSC homing mechanism consists of five different steps, namely, (1) tethering and rolling, (2) activation by cytokines, (3) cell arrest by integrins, (4) transmigration, and (5) migration.

Homing of MSCs could happen either systemically or nonsystemically. In nonsystemic homing, MSCs are transplanted directly at the target tissue and subsequently guided by chemokines and other factors to the site of injury. In contrast, in systemic homing, MSCs are administered away from the site target tissue or site of injury. Scientist proposed that the systemic homing of MSCs results from their interaction with endothelial cells in a leukocyte-like, multistep cascade which eventually allows them to migrate across the BBB. In general, several different mechanism models proposed that MSCs travel through the circulatory system and subsequently leave the blood circulation by integrating into the endothelium, transmigrating through the endothelial barrier, and penetrating the basement membrane before invading the tissue via the formation of plasmic podia [28]. The multistep cascade of MSC homing mechanism as described by Ullah et al. ([29]) consists of five different steps, namely, (1) tethering and rolling, (2) activation by cytokines, (3) cell arrest by integrins, (4) transmigration, and (5) migration, and is illustrated in Figure 1. Liu et al. [20] discussed a similar but simplified homing mechanism, which includes rolling, adhesion, and transmigration.

MSCs first enter the bloodstream when they are therapeutically administered. The homing process begins with MSCs decelerating and coming into contact with the endothelial wall by tethering to the selectins expressed by endothelial cells and starts to roll along the vasculature wall [30, 31]. Next, integrin receptors, like VLA-4, is activated in response to G-protein-coupled chemokine receptors such as stromal cell-derived factor-1 (SDF-) 1, which binds ligands expressed by MSCs such as CXCR4 or CXCR7 [29, 30]. Following activation of integrin, MSCs arrest on the endothelial membrane as integrins like VLA-4 expressed by MSC bind with VCAM-1 on endothelial cells [32]. The earlier activation increases the affinity of integrins essential for cell arrest; hence, the VLA-4/VCAM-1 interaction allows MSCs to adhere to the endothelial cells firmly [29, 32]. Next, MSCs travel through the endothelial cell layer and basement membrane in a process called transmigration, facilitated by the secretion of matrix metalloproteinases (MMPs) which degrades the endothelial basement membrane [28, 33, 34]. Alternatively, abolishment and splitting of the tight junctions between endothelial cells by MSC could also facilitate their transmigrations [24]. MSCs have also been reported to penetrate the endothelia via plasmic podia [28, 34]. Finally, MSCs migrate to the site of injury, guided by various signals released by the damaged tissue, such as growth factors and chemokines [29, 35]. Once at the target side, MSCs can induce modification of the damaged tissue microenvironment to promote regeneration and protection [36].

The delivery method is thought to influence the homing ability of stem cells. Direct transplantation at the site of injury, for example, through intracerebral injection or using microcannula, is thought to be a more efficient approach for a successful homing of the cell, although they may cause injury at the site of injection [37]. On the other hand, indirect delivery, especially through intravenous injection, has been reported to cause MSCs to be entrapped in the lung vasculature [38]. Nevertheless, over time, these MSCs eventually migrate from the lungs to other tissues, including the brain [25, 32]. However, entrapment in the lung may shorten the therapeutic life and potential effect of MSCs [39]. Literature suggests that intramuscular injection is becoming a popular method of MSC delivery, with promising results seen in the treatment of several paediatric neurological disabilities including muscular dystrophy and cerebral palsy [40]. In contrast to the short therapeutic life of MSCs delivered via intravenous injection, intramuscular injection of MSCs could potentially treat both distant or systemic conditions with sustained benefit owing to the longer dwelling time of active secretory cells and has been clinically proven to be safe [39, 41]. Alternatively, stem cells may be delivered via intranasal application, a cheap and easy alternative route found to be effective in bypassing the BBB for the treatment of neurodegenerative disorders [42, 43]. However, to this day, there is still no consensus on how stem cells used in the treatment of neurodegenerative disease should be delivered.

## 3. Stem Cell Therapy as a Treatment Modality for Neurodegenerative Diseases

The integrity of the BBB, influenced by disease severity and duration, is believed to contribute towards the progression of certain neurodegenerative diseases, although their mechanism remains unclear [44]. However, studies have shown that stem cell therapy represents a promising treatment modality in tissue regeneration and repair for many central nervous system or neurodegenerative diseases [45]. The use of stem cells like MSCs or neural stem cells has been showing promising results, with potential to slow down and, in some cases, reverse the progress of some of these neurodegenerative diseases [46–49]. Treatment with MSCs, in particular, has been very popular, likely due to their neuroprotective and immunomodulatory properties in which neurotrophic and growth factors are released by MSCs to promote tissue repair and regeneration [50]. As discussed in the previous section, stem cells are capable of homing back to the side of injury to initiate endogenous repair. Furthermore, MSCs are thought to mediate multiple mechanisms of actions, making them useful in the treatment of a wide range of diseases [51]. Interestingly, transplantation of MSCs showed the absence of cell replacement evidence, suggesting transient recovery may be induced by trophic effects [22, 36]. The application of MSCs has been extensively studied both in many animal models and some clinical studies, including but not limited to Parkinson's disease (PD), Alzheimer's disease (AD), amyotrophic lateral sclerosis (ALS), multiple sclerosis, and stroke [48]. Nevertheless, stem cells as a therapeutic tool against neurodegenerative disease comes with many benefits and challenges as summarised in Table 1.

PD is a neurodegenerative disease characterised by loss of dopaminergic cells in the substantia nigra, although it may also be caused by degeneration of other neurotransmission systems [21]. Hence, treatments that prevent the loss of dying neuronal cells or transplantation of cells with neuronal properties are imperative in managing PD [52]. Studies of the PD in both human and animal models have shown that MSCs could reverse parkinsonian symptoms [46, 47]. While the exact mechanism behind the improvement is unclear, the authors postulated that it is likely that better dopaminergic regulation could have resulted from the survival and functioning of transformed dopaminergic neurons and their terminals [47]. Follow-up of up to 36 months in the same small clinical study, in which autologous bone marrow-derived MSCs were transplanted by stereotaxic surgery, suggests that treatment with MSCs is relatively safe, without signs of tumour formation or other adverse side effects [47]. Another study suggests MSCs may have a neuroprotective effect on dopaminergic neurons via anti-inflammatory action, which promotes recovery of BBB integrity [53]. In addition, research also suggests MSCs could stabilise the permeability of the BBB by modulating astrocytic endfeet and vascular endothelial growth factor A (VEGF-A) signalling in patients with PD [44].

AD is the most common form of dementia, with currently no definite cure [54]. This disorder is pathologically characterised by the deposition of amyloid-*β* peptide and the formation of neurofibrillary tangles in affected brain regions [55]. The efficacy of MSCs in treating AD has been demonstrated in preclinical models in several studies. The feasibility of using bone marrow-derived MSCs as a therapeutic agent in an acutely induced AD mouse model has been tested by injecting MSC into the dentate gyrus of the hippocampus of the mouse [56]. The study concluded that the bone marrow-derived MSCs could increase microglia activation and promote the reduction of amyloid-*β* peptide in the brain of the AD model. In an AD-related environment, autophagy is essential as it plays a critical role in maintaining neuronal homeostasis [57]. MSCs were found to enhance autophagy and exerted a neuroprotective effect by modulating amyloid-*β* clearance in the AD mouse model. This finding suggests that a damaged AD brain could potentially be repaired by using MSCs through the modulation of the autophagy pathway [58]. The safety and tolerability of MSCs as a treatment for AD have been affirmed in phase 1 clinical trial. In the clinical trial, umbilical cord blood-derived MSCs were delivered via intraparenchymal administration in nine patients with mild-to-moderate AD. However, the efficacy of the treatment was not established due to the small sample size [59].

ALS or known as Lou Gehrig's disease is a neurological disorder with no known cure, but stem cell therapies, particularly MSCs, are a promising candidate for ALS treatment. Preclinical work in mouse models of ALS suggests that bone marrow-derived MSCs can be induced to secrete neurotrophic factors (NTFs) which delay motor neuron degeneration and improve motor performance [60, 61]. Based on this data, intramuscular (IM) implantation of bone marrow MSC-NTFs in ALS patients has been pursued in a phase 1/2 (NCT01051882) and 2a (NCT01777646) clinical trials [62]. In the phase 1/2 of the trial, six ALS patients in early stages were administered with MSC-NTFs via the IM method and another six patients with advanced stages of ALS were injected intrathecally (IT). The phase 2a clinical trial, which is a dose-escalating study, involved a total of 14 early-stage ALS patients who received a combination of IM and IT transplantation of autologous MSC-NTFs. IM implantation of MSC-NTFs in an ALS rat model study was previously shown to ameliorate motor neuron loss which occurs in the initial stage of ALS [63]. Meanwhile, MSCs which are injected through IT implantation have a greater chance of migrating to the proximity of the CNS lesions [64]. In the clinical trial, no serious adverse effects were reported following the IM, IT, and IM+IT transplantation of MSC-NTFs although some patients experienced headache, fever, vomiting, leg and back pain, and neck stiffness. Other than that, no infection nor tumour formation was found on the site of injection. Although IM implantation of MSC-NTFs only induced a minor beneficial clinical effect compared to IT implantation, both methods are safe with indications of possible, clinically meaningful benefits in patients with ALS [62].

Traumatic brain injuries (TBI) have been found to trigger neurodegeneration, which elevates the risk of developing into a more serious condition such as chronic traumatic encephalopathy, dementia, AD, and PD if left untreated [8, 65]. Increasing evidence supporting the efficiency of using MSCs in alleviating TBI sequelae has begun to emerge in recent years, suggesting that MSCs could enhance the function of a patients' nervous system. Anbari et al. have demonstrated that the intravenously administered MSCs in rats with TBI were able to differentiate into neuron- and astrocyte-like cells which then improve sensory and motor function and enhance neural growth and regeneration [66]. Another study conducted by Cox et al. has intravenously implanted MSCs into 10 children that had TBI with a Glasgow Coma Scale (CGS) score between 5 and 8 and monitored them for 6 months [67]. Among the 10 children, 7 of them showed positive results with improvements on the GCS. Another 3 children did not show significant improvement in their quality of life. However, none of them suffered from any adverse effects or died due to the use of MSC therapy. Subsequently, MSCs were used to treat TBI in adults with positive results [68], proving MSC-based approaches could serve as treatments for patients who are suffering from TBI.

## 4. Mesenchymal Stem Cells as Delivery Vehicles for Antitumour Agents

Besides their endogenous therapeutic properties, there have been increasing interest in the potential of MSCs to be used as a delivery vehicle. In this review, we will be focusing on the potential of MSCs as a drug delivery vehicle for antitumour agents, especially by leveraging on the tumour-homing ability of MSCs. Besides its inherent tumour-tropic property, MSCs are also a desirable delivery vehicle due to their immune-modulatory capabilities [30]. Several types of antiglioma agents have been studied as MSC cargoes such as prodrug enzyme, secreted proteins, oncolytic viruses, and nanoparticles.

Administration of MSCs expressing prodrug-converting gene is a promising experimental approach in glioblastoma treatment. For this purpose, MSCs are first genetically modified to express a prodrug-converting enzyme. Upon injecting back into the body, the MSCs will migrate towards the tumour cells. Subsequently, when a prodrug is administered, it will be converted into its active cytotoxic form by the enzyme expressed by the genetically modified MSCs. This in turn produces a bystander effect, causing the death of the stem cells and concurrently killing the surrounding tumour cells [69–71]. In this strategy, the homing ability of MSCs allows it to deliver a local high-dose active chemotherapeutic agent to the tumour without causing systemic toxicity which is observed in many chemotherapeutic agents [72]. Currently, thymidine kinase/ganciclovir (TK/GCV) and yeast cytosine deaminase/5-fluorocytosine (yCD/5-FC) are the most widely used enzyme/prodrug system in the literature. For instance, herpes simplex virus thymidine kinase (HSV-tk) prodrug-activating gene therapy approaches have been employed in phase III clinical trial to study its therapeutic effects in glioblastoma multiforme (GBM) patients. However, the study reported no significant differences in terms of survival rate between the treated group and the control group, which may be caused by a poor rate of delivery of the HSV-tk gene to tumour cells [73].

MSCs can also be engineered to secrete protein either through transduction with a replication-incompetent viral vector carrying gene encoding protein or transfection with a gene-carrying plasmid [31]. The gene will be transcribed and translated into protein and subsequently secreted from the MSC to affect the tumour. In this case, the engineered MSC acts as a “pump” of the secreted protein within the tumour [74]. Cytokines such as interleukin are an example of secreted proteins that have been successfully delivered by MSCs in animal glioma model systems. For instance, a preclinical study in a mouse model showed that MSCs integrated with interleukin-2 were able to migrate towards the periphery of the glioma after two weeks of MSC intracerebral injection [75]. Besides interleukin, another study showed that human MSCs secreting IFN-beta were capable of homing to gliomas following intravascular injection [76].

Additionally, MSCs can be used to deliver oncolytic viruses to gliomas. As the MSCs home to the tumour, they are capable of concealing the viruses from the immune system, although the exact mechanism is not fully understood [77]. Once loaded inside the MSCs, the virus undergoes replication and will infect and destroy the tumour cells upon release [77, 78]. Furthermore, MSCs loaded with a novel oncolytic virus named Delta-24-RGD (also known as DNX-2401) have been shown to selectively localise to the glioma cell, resulting in improved tumour eradication in subsets of mice [79]. The virus is currently being studied in numerous phase 1 and 2 clinical trials for patients with recurrent GBM; hence, results regarding its safety and efficacy are largely unpublished yet [80].

Researchers have also attempted to home MSC-bearing nanoparticles to tumour tissue in the brain. Nanoparticles have several limitations when they are administered systemically as they are diluted upon penetrating the BBB, resulting in their low concentration in the brain and have unfavourable pharmacokinetic properties [81]. Hence, the integration of nanoparticles into the MSCs is believed to circumvent this problem. A study by Roger et al. [82] has shown that polylactic acid nanoparticles and lipid nanocapsules efficiently fused into the MSCs without affecting the viability and differentiation of the cells and subsequently migrate towards human glioma xenografts. In another study, Wang et al. [83] reported that MSCs loaded with paclitaxel-poly(lactic-co-glycolic acid) nanoparticles were able to induce tumour cell death in orthotopic glioma rats with a little effect on the MSC migration capacity, cell cycle, or multilineage-differentiation potential.

Despite the promising potential of MSC-mediated delivery of antiglioma agents, there are still challenges that need to be overcome. Firstly, the modification of MSCs bearing antitumour agents should not alter its tumour-homing capacity [30]. It is also important to put into consideration that different sources of MSCs may have different abilities in promoting or suppressing the growth of glioma cells under different conditions. In addition, the cargoes must not prematurely kill the MSC and should only be released from the MSC once within the tumour [72]. Further studies are highly required as there is still much discrepancy in terms of the efficiency of the combination of these antiglioma agents and MSCs. Lastly, a better understanding of the biological consequences of using MSC as delivery agents for antiglioma agents is of utmost importance before it can be widely applied for treating patients with malignant gliomas.

## 5. Conclusion

Treatment with stem cells thus far presents promising potential in treating patients with neurodegenerative diseases, with many clinical safety and efficacy experiences. The affinity and ability of stem cells to bypass the BBB and migrate to the brain makes them a versatile treatment modality, either by relying on the endogenous therapeutic properties of stem cells or by leveraging on their homing property for drug delivery purposes. Nevertheless, more research is necessary to understand the underlying mechanism of action better and to broaden the application of stem cells as a therapeutical tool in the treatment of neurodegenerative diseases.

## Figures and Tables

**Table 1:** Benefit and limitation of stem cells as a therapeutical tool in neurodegenerative diseases.

Benefit	Limitation
(i) Possess neuroprotective and immunomodulatory properties which promote tissue repair and regeneration	(i) Underlying mechanism of action remains unclear and will require more detailed investigations
(ii) Capable of migrating across the endothelial cell of BBB	(ii) Must successfully overcome the BBB in order to exert therapeutic effects in the brain
(iii) Capable of homing back to the side of injury to initiates endogenous repair	(iii) Homing potential is influenced by the delivery method
(iv) Absence of cell replacement evidence; trophic effects likely induce transient recovery	(iv) Sufficient numbers of cells reaching the target site are necessary to exert a therapeutical effect
(v) Generally well-accepted with no serious adverse effects such as infection or tumour	(v) Treatment outcome may be affected by various factors including the donor's age, host tissue, and growth regulators expressed by recipient tissue
